# USE OF TECHNOLOGY BY FOUR DIVERSE COHORTS OF OLDER ADULTS: FINDINGS FROM THE CART STUDY

**DOI:** 10.1093/geroni/igz038.1196

**Published:** 2019-11-08

**Authors:** Katherine Wild, Nora Mattek, Nicole Sharma, Jennifer Marcoe, Rachel Wall, Jeffrey Kaye

**Affiliations:** 1 OHSU, Portland, Oregon, United States; 2 Oregon Health & Science University, Portland, Oregon, United States

## Abstract

Early studies of technology adoption and computer use identified a “digital divide” between older adults and the general population. As that gap has narrowed, other demographic variables have been identified as continuing to foster disparities in access to and use of computers and related technologies. For example, gender, socioeconomic status, education, and ethnicity have been recognized as predictors of computer use among community living older adults. The ORCATECH Collaborative Aging (In Place) Research Using Technology (CART) initiative was designed to develop and validate an infrastructure for research utilizing technologies to facilitate healthy and independent aging. The CART program tests innovative technology applications in four diverse populations: residents in low income, section 202 housing in Portland; isolated, rural veterans in the Pacific Northwest; urban African American seniors in Chicago; and socially isolated, ethnically diverse low income seniors in Miami. As part of their participation in the CART project, older adults complete an annual survey of health and technology use. A total of 214 participants were enrolled and agreed to have their homes instrumented with the CART platform of monitoring technologies. Across all four cohorts 166 answered the technology survey thus far: 82 - 97% of participants own a cell phone; 64 - 78% perform some online banking activities. There were no differences among cohorts in computer use or cell phone ownership, or in other measures of technology use. Inclusion of ethnically and economically diverse populations in future technology research will be critical in the development of effective digital health interventions.

